# Thyroid-Originating Extracellular Vesicles Harbor Thyroid-Specific Biomarkers with Potential Relevance for Thyroid Cancer Recurrence Detection

**DOI:** 10.3390/ijms27083510

**Published:** 2026-04-14

**Authors:** Nevena Bobar, Ninoslav Mitić, Maja Kosanović, Sonja Šelemetjev, Tijana Išić Denčić, Katarina Taušanović, Jelena Janković Miljuš

**Affiliations:** 1 Institute for the Application of Nuclear Energy—INEP, University of Belgrade, Banatska 31b, 11080 Belgrade, Serbia; nevena.bobar@inep.co.rs (N.B.); ninoslavm@inep.co.rs (N.M.); maja@inep.co.rs (M.K.); sonja@inep.co.rs (S.Š.); tijana@inep.co.rs (T.I.D.); 2 Clinic for Endocrine Surgery, University Clinical Center of Serbia, Pasterova 2, 11000 Belgrade, Serbia; katarinatausanovic@gmail.com; 3 Faculty of Medicine, University of Belgrade, Doktora Subotića 8, 11000 Belgrade, Serbia

**Keywords:** extracellular vesicles, thyroid cancer, recurrent thyroid cancer, thyrotropin-receptor, TSHR, thyroglobulin, thyroid cell lines, human plasma

## Abstract

Thyroid cancer (TC) is the most common endocrine malignancy, and challenges persist in preoperative diagnosis of indeterminate nodules and postoperative monitoring when thyroglobulin (Tg) assays are compromised by interfering anti-Tg antibodies (Tg-Ab). Extracellular vesicles (EVs) carry molecular cargo reflective of cells of origin and are increasingly explored as biomarker sources. In this study, we investigated whether thyroid-derived EVs retain the expression of thyroid-specific thyrotropin-receptor (TSHR), a suitable target in immunoaffinity-based EV isolation, and explored the presence of Tg in EV cargo as potential surrogate for serum Tg. EVs from thyroid cell lines (Nthy-Ori 3-1, TPC-1, OCUT2) and plasma of patients with benign, malignant tumors and recurrent TC were isolated by differential ultracentrifugation and characterized via nanoparticle tracking and Dot and Western blot analyses. EVs derived from Nthy-Ori 3-1 and TPC-1 cell lines were positive for surface TSHR and vesicular Tg, but not OCUT2. All plasma-derived EVs were positive for TSHR and Tg, while their electrophoretic profiles from vesicles differed compared to tissue lysate. Tg was detectable in EVs isolated from recurrent TC samples, even in Tg-Ab positive cases. Together, these results support the use of TSHR for targeted EV isolation and point to vesicular Tg as a potential recurrence marker.

## 1. Introduction

Thyroid cancer (TC) is the most common endocrine malignancy whose incidence is increasing worldwide in all age groups, with a 30% projected rise by 2030 [1]. It presents as a thyroid nodule, with the decision of whether to surgically remove the lesion being made via a cytological analysis of material gathered with fine needle aspiration biopsy (FNAB). However, the analysis is unreliable for 30% of samples, which ultimately receive surgical treatment irrespective of the nature of the tumor [2]. In half of those cases, this leads to unnecessary surgery of benign nodules, causing a lower quality of life for patients and higher costs for healthcare. Meanwhile, cases of differentiated thyroid cancer are successfully treated with surgery and radiotherapy. However, recurrence appears in 10–30% of cases, with a significant proportion of late recurrences as 20% recur after the 10-year mark, thus warranting a reliable follow-up test [3,4]. Monitoring for recurrent disease is performed with measuring serum thyroglobulin (Tg) levels, but it is not without obstacles—the appearance of anti-Tg (Tg-Ab) and heterophile antibodies interferes with immunometric Tg tests [5,6]. Recently there have been efforts in upgrading the immunoassays for Tg detection methods to solve the issue of interfering antibodies by modifying the preanalytical processing of the samples [7,8], bringing back the issue of Tg detection in differentiated thyroid cancer (DTC) recurrence to the spotlight. Still, the quest for reliable early diagnostic markers and surrogate markers for follow-up of patients harboring interfering immunoglobulins is ongoing.

Extracellular vesicles (EVs) have recently been recognized as potential sources of cell-specific biomarkers, and can be isolated from various bodily fluids [9]. They represent lipid bilayer-encircled subcellular structures secreted by most cells, with cell-to-cell communication purposes, and carry molecular cargo that reflects both the identity and the state of the cell of origin [10]. Subsequently, the pathological processes in the tissue of origin can be recapitulated in the vesicular cargo and EV surface, which ultimately makes EVs attractive potential sources of disease biomarkers or therapeutic response markers [11]. Their stability in the circulation and other bodily fluids enables their use in liquid biopsies [12].

A number of studies have investigated the potential of EV cargo in diagnosing thyroid cancer, with promising results [13,14]. Most studies have explored the diagnostic potential of non-coding RNA species from EVs (microRNAs, circular RNAs, long non-coding RNAs). An NGS profiling study of plasma EVs identified 129 miRNAs differentially expressed between malignant nodules and benign goiter, with some AUCs reaching ~0.95 [15]. Moreover, miR-146b-5p and miR-21a-5p in EVs differentiated papillary thyroid carcinoma (PTC) from benign disease more reliably than total plasma miRNAs in a preoperative cohort [16]. On the protein side, HSP27, HSP60, and HSP90 were significantly elevated preoperatively in plasma EVs of PTC patients, further illustrating the potential of EV-based liquid biopsy. One study to date examined the use of Tg from EVs in thyroid cancer follow-up; however, it used vesicles from urine, rather than circulation [17]. Although these markers are promising, their use is still hindered from clinical applications due to the lack of standardization of pre-analytical and analytical procedures.

Among the reasons hampering the standardization of circulating EV analysis is the scarcity of tissue-specific vesicles obtained from blood in total vesicular isolates [18]. One way to overcome this is by enriching the EV preparations with tissue-specific EVs by using immunoaffinity-based purification methods [19]. The necessary step for constructing such a method is to define the presence of tissue-specific biomarkers on the surface of EVs that can act as immunoaffinity targets. In the case of thyroid-derived EVs, the presence of thyroperoxidase (TPO) has been confirmed and used for enriching EV preparations from blood [20]. The presence of another thyroid-specific protein, thyrotropin receptor (TSHR), has been detected on the surface of EVs derived from TC cell lines [21]. To the best of our knowledge, the detection of TSHR has not been performed on the samples of EVs isolated from the circulation of patients harboring thyroid tumors.

The aim of our study was to further explore the content of thyroid-originating EVs, with the goal of defining potential immunoaffinity-surface targets, as well as thyroid-specific biomarkers of disease. We postulated that surface receptors for thyrotropin will be present on thyroid-originating EVs, thus presenting an exploitable target for immune-isolation methods of EVs. Secondly, we hypothesized that thyroid-originating EVs will have enclosed Tg (vesicular Tg) that has the potential to be a surrogate marker for thyroid cancer recurrence monitoring in Tg-Ab positive patients. We tested these hypotheses firstly on human thyroid cell lines, as sources of exclusively thyroid-derived vesicles, and continued the investigation with EVs isolated from human plasma of patients harboring benign and malignant thyroid nodules, including recurrent thyroid cancer cases several of which were positive for Tg-Ab.

## 2. Results

### 2.1. Characteristics of EV Population

**Figure 1:**
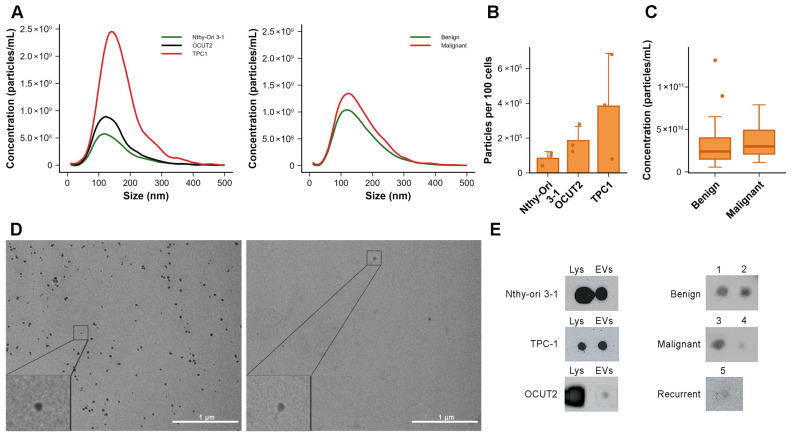
Characterization of EVs from conditioned medium of thyroid cell lines and plasma of thyroid nodule patients via NTA and TEM analyses. (**A**) Size distribution of particles in EV enriched preparations determined by NTA derived from thyroid cell culture (left) and plasma of patients with thyroid tumors (right). Graphs represent mean values of all measured samples (*n* = 3 for each cell line, and *n* = 82 for plasma samples). (**B**) Number of EVs derived from thyroid cell culture (*n* = 3 for each cell line) determined by NTA, represented as the number of EVs per 100 cells. Bars represent mean ± SD, dots represent single measurements. (**C**) Concentration of EVs isolated from the plasma of thyroid tumor patients determined by NTA (*n* = 82). Box-plots represent the IQR with the median and whiskers following Tukey’s rule. (**D**) TEM micrographs of particles with cup-shaped morphology and up to 100 nm diameter in EV preparations from Nthy-Ori 3-1 thyroid cell culture (left) and plasma of a patient with benign thyroid tumor (right). (**E**) Representative images of dot blot analysis of CD63 presence in lysates of thyroid cell lines and their EVs (left); and EVs derived from the plasma of patients with benign, malignant and recurrent thyroid tumor cases, numbers denote individual case samples (right). Lys-thyroid cell lysate. CD63 was analyzed using intact EVs. EV—extracellular vesicle.

Nanoparticle tracking analysis was used to determine the concentration of vesicles in the pellet obtained after differential ultracentrifugation, and to assess their size distribution. Analysis of samples of isolated EVs from conditioned cell culture medium confirmed the presence of EVs with median diameters ranging from 100 to 190 nm (representative size distribution plots are shown in Figure 1A, left panel). The average EV diameter did not significantly differ between the cell lines. EVs isolated from the conditioned medium of Nthy-Ori 3-1 cells had a mean particle diameter of 136.7 ± 15.9 nm, while EVs from TPC-1 and OCUT2 cells measured 146.0 ± 6.7 nm and 132.1 ± 6.3 nm, respectively (mean ± SD of median diameters, *n* = 3 in each group). Particle concentrations measured by NTA showed modest differences between cell lines, with median EV concentrations of 1.8 × 10^10^ particles/mL (range 0.7–2.0 × 10^10^) for Nthy-Ori 3-1, 4.7 × 10^10^ particles/mL (range 0.9–8.2 × 10^10^) for TPC-1, and 1.4 × 10^10^ particles/mL (range 1.1–2.5 × 10^10^) for OCUT2 cells. After normalization to cell number, TPC-1 cells exhibited the highest EV release, producing approximately two-fold more EVs per cell than OCUT2 cells and approximately 4.6-fold more EVs per cell than Nthy-Ori 3-1 cells (Figure 1B).

In the same way, we determined the size and number of isolated EVs originating from the plasma of patients with thyroid nodules (*n* = 82); representative size distribution plots are shown in Figure 1A, right panel. The average diameter of the isolated EVs was 144.5 ± 16.6 nm for the group of benign samples, and 145.2 ± 15.1 nm for the malignant group (mean ± SD of median diameters). No difference was observed in diameter between EVs originating from benign and malignant thyroid nodules (Student’s *t*-test, *p* = 0.748). The median number of EVs in the benign group was 2.4 × 10^10^ (range 0.55–14.0 × 10^11^), while the median EV number in the malignant group was 2.9 × 10^10^ (range 0.66–7.9 × 10^10^, Figure 1C). The number of EVs isolated from the plasma of patients with malignant thyroid nodules was approximately 1.2× higher compared to the number of EVs isolated from the plasma of patients with benign nodules, but without a statistically significant difference (Mann–Whitney U test, *p* = 0.157).

To confirm that the method used for EV isolation (dUC) enables the extraction of small EVs and to corroborate NTA results, selected samples of EV-enriched preparations isolated from conditioned medium (Nthy-Ori 3-1 cell line derived EVs), as well as from patient plasma (case with benign thyroid tumor) were imaged using transmission electron microscopy. It is important to note that due to the volume restrictions of the sample, we were able to perform TEM only in size-exclusion (SEC) purified plasma EVs. This was the initial way of EV purification, used in the optimization phase. TEM imaging confirmed the presence of a heterogeneous population of round, membrane-enclosed vesicles, with a diameter of up to 100 nm originating from both conditioned medium and plasma-derived EVs (Figure 1D). However, a low number of particles were visualized on TEM in the plasma samples, due to the additional SEC purification step. As this would not render sufficient EV protein concentrations for Western blot, the second purification step was not used in further experiments.

The presence of the vesicle-specific marker CD63 on EV surface was assessed using the dot blot method for the purpose of qualitative characterization of EV surface markers. In this analysis, vesicles isolated from the conditioned medium of three cell lines and from representative plasma samples of five patients (2 benign, 2 malignant, 1 recurrent case) were used. CD63 was detected on the surface of EVs derived from all three cell lines, as well as in the corresponding cell lysates (Figure 1E, left panel). CD63 was also detected in EVs isolated from patient plasma samples, including those from individuals with benign, malignant, and recurrent thyroid tumors (Figure 1E, right panel). Combined results from TEM, NTA, and CD63 dot blot analysis confirm that the differential ultracentrifugation method enables the isolation of vesicles consistent in size, morphology, and surface marker profile with small EVs.

### 2.2. Analysis of the Presence of TSHR on EV Surface and Tg in EV Cargo of Thyroid Cell Culture-Derived EVs and Thyroid Nodule Patient Plasma EVs

**Figure 2:**
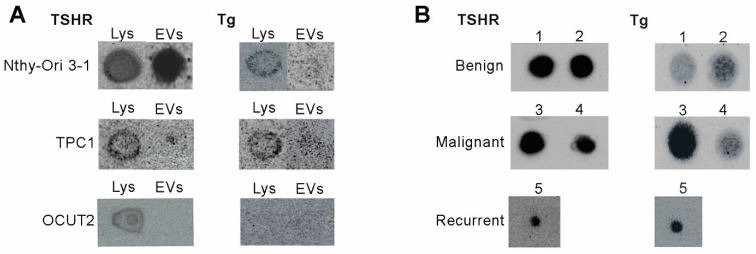
Representative images of dot blot analysis of thyroid-specific proteins TSHR and Tg presence in (**A**) EV-enriched preparations derived from normal and cancer thyroid cell lines (*n* = 3 for each cell line), as well as their cell lysates (Lys). (**B**) EV-enriched preparations derived from the plasma of patients with benign, malignant and recurrent thyroid tumors (*n* = 5); numbers denote individual case samples. TSHR was analyzed using intact EVs, while Tg was analyzed in SDS-treated EVs. EV—extracellular vesicle, TSHR—thyrotropin receptor, Tg—thyroglobulin.

The presence of TSHR on the surface of EVs and Tg within the vesicular cargo was assessed by dot blot analysis in EVs derived from cell lines and from representative plasma samples of patients (2 benign, 2 malignant, 1 recurrent case). Dot blot analysis was performed as a qualitative assessment of protein markers of EVs, as it provides a rapid screening method, whereby samples are concentrated in a single spot, which can facilitate antibody detection, especially in the cases of surface EV proteins. TSHR was detected in the cell lysates of all analyzed cell lines, as well as on the surface of EVs from Nthy-Ori 3-1 and TPC-1 cell lines. Although OCUT2 cell lysate was positive for TSHR presence, albeit in lower amounts than the other two cell lines, we did not detect the receptor on the surface of EVs (Figure 2A, left panel). The results of dot blot analysis using EVs derived from patient plasma showed that TSHR is present in all analyzed EV preparations (Figure 2A, right panel).

To detect vesicular Tg EVs were treated with SDS detergent in order to release their cargo. Dot blot analysis of vesicular Tg from thyroid cell lines showed that EVs from Nthy-Ori 3-1 and TPC-1 cell lines contain Tg. Consistent with this, their cell lysates were also positive for the thyroid-specific protein. On the other hand the presence of Tg was not detected in EVs from OCUT2 cell lines, and this was consistent with the result from OCUT2 cell lysates which were also Tg-negative. (Figure 2B, left panel). The samples of the patient’s plasma were positive for the presence of vesicular Tg, while, importantly, Tg was also detectable in the EVs isolated from the plasma of patients with recurrent thyroid cancer (Figure 2B, right panel).

### 2.3. Levels of TSHR and Tg from Thyroid-Originating EVs in Benign and Malignant Thyroid Nodules

**Figure 3:**
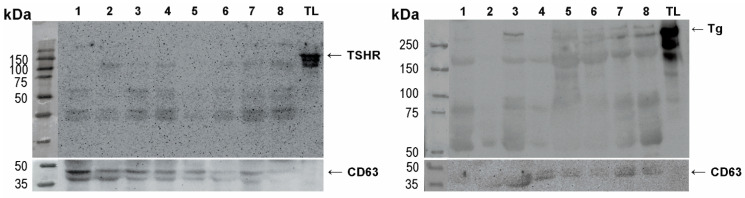
Representative Western blot images for TSRH (**left**) and Tg (**right**) analyses in EVs. The proteins were detected in EV preparations isolated from the plasma of patients with benign (cases 1–4) and malignant (cases 5–8) thyroid tumors. Thyroid tissue lysate (TL) was used as a positive control; CD63 was used as a loading control. The arrow for TSHR is pointing to the band at the expected molecular weight for intact TSHR (~120 kDa), and the arrow for Tg points out to the band at expected MW for a Tg monomer (~330 kDa). EV—extracellular vesicle, TSHR—thyrotropin receptor, Tg—thyroglobulin.

**Figure 4:**
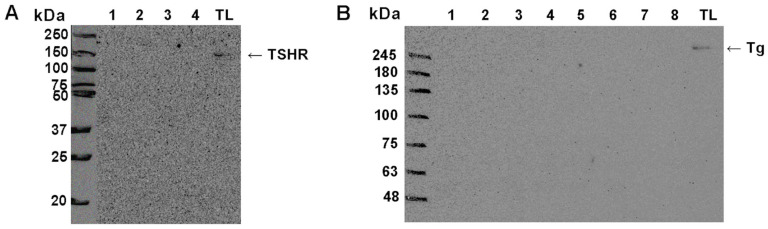
Western blot analysis of TSHR and Tg presence in supernatants remaining after dUC enrichment of extracellular vesicles from human plasma. (**A**) TSHR was analyzed in cases with benign (1, 2, 4) and malignant (3) thyroid tumors. Arrow for TSHR is pointing to the band at the expected molecular weight for intact TSHR. (**B**) Tg was analyzed in cases with malignant (1), recurrent (2, 3, 4, 5) and benign (6, 7, 8) thyroid tumors. Arrow for Tg pointing to the band at the expected molecular weight for Tg monomer. For both analyses, the amount of supernatant loaded to the gel corresponded to the total protein plasma concentration of 0.7 µg, compatible to approximated EV protein concentration for the number of EVs used for Western blot normalization. Thyroid tissue lysate (TL) was used as a positive control. EV—extracellular vesicle, TSHR—thyrotropin receptor, Tg—thyroglobulin, dUC—differential ultracentrifugation.

To better understand the diagnostic potential of these markers, we analyzed the expression of TSHR and Tg in a larger number of samples of EVs isolated from the patient’s plasma. For this analysis, we employed Western blot, rather than dot blot, as a method that provides more information on the analyzed samples, such as the relative molecular weight of the detected proteins, and enables more specific semi-quantitative, densitometric analyses. Due to the scarcity of EV material, a limited number of experiments can be performed using each sample. We were able to successfully retrieve the results for WB protein analysis on the following number of cases for TSHR: *n* = 76, out of which 47 benign, 29 malignant; and for Tg: *n* = 62, out of which 37 benign, 25 malignant. We confirmed the presence of TSHR and Tg in all tested EVs samples, while there was a difference in the electrophoretic profile of the TSHR protein between EVs and tumor-adjacent normal thyroid tissue lysate, which was used as a positive control (Figure 3). Vesicular TSHR was detected as three distinct bands, at the approximate molecular weight (MW) of 120 kDa, 75 kDa, and 50 kDa, possibly representing different molecular forms of the receptor (full-length and A-subunits). In tissue samples, the observed bands appeared predominantly above 120 kDa. Regarding Tg, we observed bands at 330 kDa which is the expected MW for Tg monomer, and also detected bands at lower relative MW, with the most prominent ones at 200 kDa and 60 kDa. Electrophoretic profiles of Tg in EVs were different from the ones in tissue lysate, where only the most prominent bands were observed at 330 kDa and ~200 kDa, following less prominent signals on lower MW (~70 kDa). No detectable TSHR or Tg expression was observed in supernatants remaining from the plasma isolation of EVs (Figure 4).

To assess potential association of Tg species with the protein corona, EVs from two benign cases were subjected to a limited protease digestion assay. After incubating the samples with 2.5 ug/mL of the enzyme NTA was performed to ensure the particle number did not significantly decrease upon digestion, which would mean the EVs have been degraded. After establishing that EV number did not decrease (Supplementary Table S1), we performed WB analyses in nontreated vs. treated samples (Supplementary Figure S1). Proteinase K treatment selectively degraded the high-molecular weight (~330 kDa) Tg species in one sample while preserving lower-molecular weight forms, suggesting that the former may be surface-accessible and could be associated with protein corona, whereas the latter may be protected within intact vesicles.

We used densitometric analysis to quantify the relative protein expression of TSHR, Tg, and CD63. CD63 was used as a loading control for EVs; however, it is not expressed in all vesicles, but predominantly in an exosome-like vesicle subpopulation. While this subpopulation likely constitutes the majority of EVs present in pellets obtained using this isolation method, CD63 cannot be considered representative of all EV species. Therefore we performed the analysis of vesicular TSHR and Tg expression by normalizing samples to: (i) particle number determined by NTA, by which the results present the expression of TSHR and Tg in total EV pellet; and (ii) particle number determined by NTA and, additionally, to CD63 densitometric values, by which the results represent TSHR and Tg expression in the subpopulation of CD63-positive vesicles. Due to the limited availability of EV material, CD63, used as a normaliser, could only be assessed in 35 cases for TSHR and 32 cases for Tg. All detected bands for both proteins were measured and compared between benign and malignant samples. The expression of CD63 did not differ significantly between benign and malignant samples (Mann–Whitney U test, *p* = 0.195; r = 0.223).

**Figure 5:**
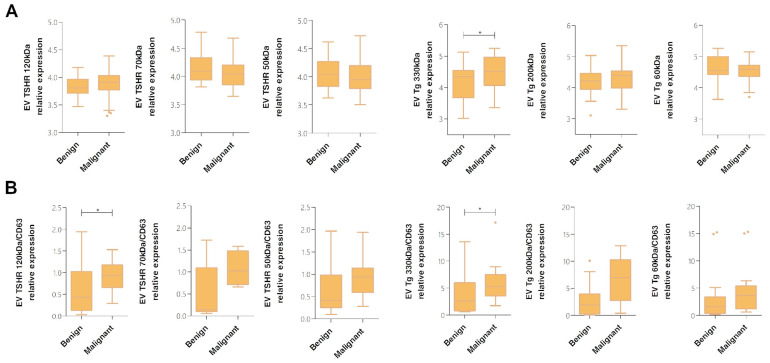
Relative expression levels of vesicular TSHR and Tg in benign and malignant samples. Densitometric measurements of TSHR was performed at band MW of 120 kDa, 70 kDa and 50 kDa; and Tg was measured at band MW of 330 kDa, 200 kDa and 60 kDa. Results present relative expression levels normalized to (**A**) total particle number determined by NTA, and (**B**) total particle number determined by NTA and CD63 vesicular expression levels. Box-plots represent the IQR with the median and whiskers following Tukey’s method. Densitometric results normalized to particle numbers only were transformed logarithmically in order to fit Gaussian distribution. * *p* < 0.05; EV—extracellular vesicle, TSHR—thyrotropin receptor, Tg—thyroglobulin.

When the expression of TSHR and Tg was analyzed in total EV pellet (normalized to particle number only), we observed that all measured bands for TSHR were not differentially expressed in benign vs. malignant samples (Student’s *t*-test, *p* > 0.05). In the case of Tg, the band at 330 kDa (EV Tg 330 kDa) was expressed at a significantly higher level in malignant than in the group of benign samples (Student’s *t*-test, *p* = 0.031, Cohen’s d = 0.569, 95% CI [−0.601, −0.029]; Figure 5A).

In the subgroup of CD63-positive EVs, we observed that both TSHR and Tg were differentially expressed between benign and malignant samples. The TSHR band at 120 kDa (EV TSHR 120 kDa/CD63) showed significantly higher expression levels in malignant than benign samples (Student’s *t*-test, *p* = 0.025), while the same trend was observed for other bands but without statistical significance. Levels of thyroglobulin bands at 330 kDa (EV Tg 330 kDa/CD63) and 200 kDa (EV Tg 200 kDa/CD63) showed a trend of higher expression in the malignant group; however, only levels of EV Tg 330 kDa/CD63 were significantly different between benign and malignant samples (Student’s *t*-test, *p* = 0.020; Cohen’s d = 0.848, 95% CI [−9.786, −0.909]; Figure 5B).

### 2.4. Detecting Vesicular Tg in the Plasma of Patients with Recurrent Thyroid Cancer

**Figure 6:**
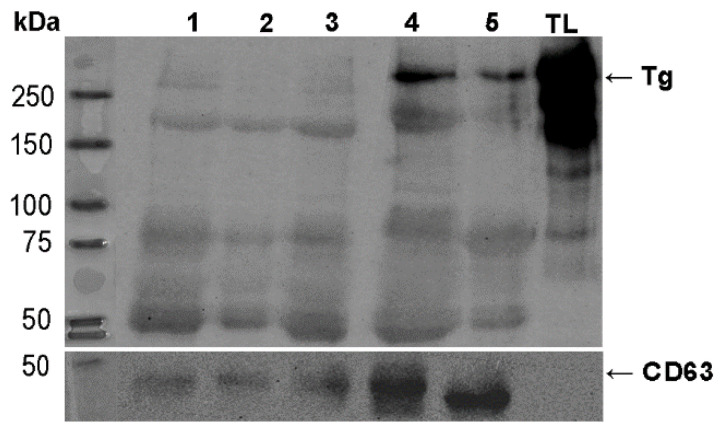
Protein levels of detected vesicular Tg from plasma EVs of recurrent thyroid cancer patients. Tg was detected in EVs from recurrent thyroid cancer cases (1–5), and tissue lysate was used as positive control (TL). Samples 4 and 5 were positive for Tg-Ab. Arrow for Tg points out to the band at expected MW for a Tg monomer. EV—extracellular vesicle, Tg—thyroglobulin, MW—molecular weight.

We tested the levels of Tg in five samples of EVs from plasma of patients harboring thyroid cancer recurrence. The clinical characteristics of this cohort are summarized in Table 1. The cohort consisted of patients presenting with locoregional lymph node metastases after initial surgery for thyroid cancer, with one case showing persistent thyroid cancer, as indicated by non-decreasing serum Tg levels after total thyroidectomy. One patient developed distant metastasis to the lungs in later follow-up. All cases were initially diagnosed with PTC as their primary tumor. The follow-up time prior to the study ranged between 5 and 366 months. Tg was detectable in all five samples of EVs with varying intensity, as well as in two samples that were positive for Tg antibodies (Figure 6). Due to a low sample size in this cohort, we performed (*n* = 5, while data for serum Tg was available in 4 cases) an exploratory analysis of the association of the levels of serum Tg, assessed with immunoassays, and EV Tg normalized to CD63 levels. The analysis showed a strong positive correlation of the two parameters (Supplementary Figure S2; Pearson’s correlation coefficient = 0.992, *p* = 0.008). Given the very limited sample size, this finding needs to be considered purely exploratory.

### 2.5. Analysis of Vesicular TSHR and Tg for Diagnostic and Prognostic Utility

**Figure 7:**
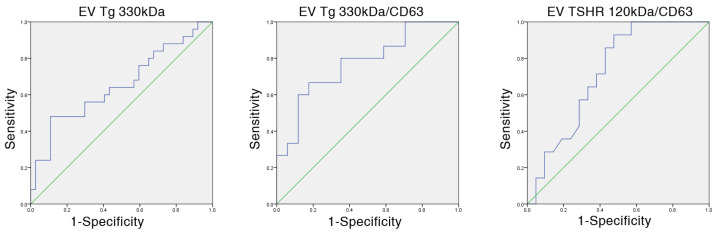
Receiver operating curves (depicted in blue) for EV Tg 330 kDa, EV TSHR 120 kDa/CD63, EV Tg 330 kDa/CD63. The diagonal line depicted in green represents the reference line corresponding to random classification.

We performed a Receiver operating curve (ROC) analysis to assess the diagnostic accuracy of vesicular TSHR and Tg for the bands that showed statistically significant difference between benign and malignant samples (EV Tg 330 kDa, EV Tg 330 kDa/CD63, EV TSHR 120 kDa/CD63). The results of the analysis are summarized in Table 2, while the ROCs where statistically significant area under the curves (AUC) was observed are presented in Figure 7. Among the tested markers, only EV Tg 330 kDa in CD63 positive vesicles had a fair AUC value (>0.7, *p* = 0.008). Cut-off values for all markers were calculated according to ROC analysis and sensitivity, specificity, positive predictive value (PPV), negative predictive value (NPV) and diagnostic accuracy (ACC) were calculated (Table 2). As expected, good sensitivity (80%) and fair ACC (>70%) was observed only for EV Tg 330 kDa/CD63. Additionally, we performed an analysis of the association of the selected markers with clinicopathological parameters of the patients used in the study (Supplementary Tables S2 and S3). Significant negative associations were observed for EV TSHR 70 kDa with tumor size (Pearson’s coefficient = −0.429, *p* = 0.037), and pathological T (pT) stage (Pearson’s coefficient = −0.582, *p* = 0.029). Association with pT was lost when a cut-off value for pT was introduced. Serum Tg levels did not correlate with any of EV Tg species. EV Tg 330 kDa normalized to CD63 showed an increasing trend according to a differentiation score of the lesion (calculated according to histology and aggressiveness) (Supplementary Figure S3; Spearman’s rho = 0.430, *p* = 0.014). The same trend was not observed for EV Tg 330 kDa total free serum Tg (Spearman’s rho = 0.197, *p* = 0.125; Spearman’s rho = −0.100, *p* = 0.488).

## 3. Discussion

Monitoring thyroid cancer recurrence is challenging in patients harboring Tg-Ab. Tissue-specific EVs can offer an alternative source of thyroid-specific biomarkers, although they require specialized and optimized enrichment methods. This study explored the molecular cargo of thyroid-derived EVs, hypothesizing that they display TSHR on their surface and enclose Tg. We established that TSHR is present on the surface of EVs originating from thyroid cell lines as well as from the plasma of patients with thyroid tumors, thereby opening a possibility of exploring it as a thyroid-specific EV marker in immuno-isolation methods. Further, we detected Tg within the cargo of these EVs, while its presence in circulating EVs from all patients with recurrent thyroid cancer, including cases positive for Tg-Ab, shows vesicular Tg can be further explored as a surrogate biomarker for recurrence monitoring.

We analyzed EVs released by three thyroid-derived cell lines representing normal thyroid epithelium, differentiated thyroid cancer, and dedifferentiated thyroid cancer, i.e., Nthy-Ori 3-1, TPC-1 and OCUT2 cell line, respectively. The NTA showed that mean EV size from cell lines is below 200 nm, corresponding to the diameter range of small EVs [22]. Probing for EV surface markers revealed the presence of the tetraspanin CD63 on EVs originating from all three analyzed cell lines. Given that plasma membrane components and associated proteins can be selectively incorporated into EV membranes during their formation either through direct budding (microvesicles) or via endosomal sorting (exosomes) [23], we anticipated that membrane-bound proteins such as TSHR could also be present on the surface of EVs derived from thyroid cells. Therefore, we first confirmed the presence of TSHR in the cell lysates of cell lines included in the study, demonstrating that each line expresses TSHR. This finding is consistent with previously published data showing that TPC-1 and OCUT2 cell lines express a functional TSH receptor [24]. With respect to TSHR expression on EVs, those released by Nthy-Ori 3-1 and TPC-1 cells were TSHR-positive, while EVs from OCUT2 cells had no detectable amounts of TSHR on their surface. Edo et al. [21] also detected TSHR on the surface of EVs derived from Nthy-Ori 3-1 cells, while the other two cell lines used in our analysis were not previously characterized. The results of our TPC-1-cell and EV surface marker analysis are consistent with the differentiated nature of this PTC-derived cell line, as well-differentiated papillary thyroid carcinomas are known to retain the expression of a functional TSHR [25]. In contrast, the absence of this receptor in EVs from OCUT2 cells may reflect the dedifferentiated phenotype of this cell line, related to the lower abundance of this protein in the corresponding cell lysates. This finding aligns with reports that anaplastic thyroid carcinoma (ATC) tissues contain markedly reduced amounts of TSHR compared to normal thyroid tissue and other thyroid carcinoma types, reflecting the loss of differentiation in this phenotype [26,27,28]. This might, potentially, affect and alter mechanisms of protein sorting into EVs [29].

In further characterization of EVs originating from thyroid cell lines, we analyzed the presence of the intravesicular, thyroid-specific protein Tg first confirming its synthesis by the selected cell lines. We found that the Nthy-Ori 3-1 and TPC-1 cell lines express Tg, whereas this was not the case for the OCUT2 cell line. Intravesicular Tg in our study was detected in EVs from Nthy-Ori 3-1 and TPC-1 cell lines, and not in OCUT2-derived EVs, which is consistent with the lack of its expression in OCUT2 cell lysate. Data from the literature corroborates our results for vesicular Tg in normal thyroid-derived cells, as Cui et al. showed that Nthy-Ori 3-1 EVs are positive for Tg [30]. In addition, the study of Vlasov et al. [31] showed that EVs of normal rat thyroid cells FRTL-2 possess Tg inside their cargo. The absence of Tg in OCUT2 is attributed to the loss of Tg synthesis in anaplastic thyroid carcinoma cells, as a consequence of their dedifferentiated phenotype [32]. The presence of Tg in EVs from the TPC-1 cell line has not been investigated to date; our results demonstrated that this protein is detectable in vesicles from TPC-1 cells, which is consistent with our finding that this cell line retains the ability to produce Tg. Previous literature data regarding Tg expression in TPC-1 cell line report the lack of Tg mRNA expression [33,34]. However, discrepancies with thyroid specific markers in cell lines are often seen and may stem from heterogeneity that can arise within a single cell line across different laboratories [35]. Given the possibility of Tg contamination from cell culture medium of cell lines that retain the production and secretion of Tg, and considering that the dUC isolation method can co-precipitate protein aggregates with EVs, we cannot completely rule out the possibility that Tg detected in EV preparations originates from secreted EV-free Tg [36]. Nevertheless, since Tg aggregates primarily form within the follicular lumen, it can be assumed that the likelihood of Tg contamination in the EV preparations is very low, as Tg aggregates would not be expected to form in a monolayer cell culture system [37]. Additionally, cell cultivation for EV production was performed without FBS, therefore eliminating the possibility of contamination from bovine Tg or bovine circulating EVs that might contain Tg.

The analysis of surface markers on EVs isolated from patient plasma revealed the presence of CD63 in the EV preparations, confirming that the selected isolation method successfully yielded a population of CD63-positive vesicles. Next, the EVs from plasma were uniformly positive for the TSH receptor. Given that the thyroid presents the predominant physiological site for TSHR expression, it is reasonable to assume that TSHR-positive EVs are primarily thyroid-derived. Although TSHR expression has been reported in other tissues, its levels are exceptionally low compared to those in thyrocytes [27]. More specifically, while only orbital fibrocytes in Graves’ ophthalmopathy show TSHR expression at levels of approximately 10–100-fold lower than the thyroid [38], TSHR expression in other non-thyroidal tissues remains 200–10,000-fold lower than thyroidal expression [39]. Moreover, studies that reported detectable TSHR gene expression in non-thyroidal tissues used high-sensitivity detection methods, which further supports the notion that TSHR is present only at extremely low levels outside the thyroid. Consistent with this, Cui et al. [40] analyzed the involvement of EVs in the pathogenesis of Graves’ disease, and this study confirmed that EVs derived from the plasma samples of patients with Graves’ disease and Graves’ ophthalmopathy were positive for TSHR. To the best of our knowledge, however, no study has evaluated the presence of TSHR on EVs derived from the plasma of patients with benign and malignant tumors. Our findings that all analyzed vesicles express TSHR on their surface present a rationale for constructing an immunoaffinity method based on vesicular TSHR immune-capture and testing the possibility of using such a method to prepare EV pellets from plasma that will be highly thyroid-specific.

Interestingly, Western blot analysis of plasma EV-associated TSHR revealed that its electrophoretic profile in EVs differs from that observed in thyroid tissue used as control. Three bands consistently appeared in all EV samples, with varying intensities, likely corresponding to different subunits or cleavage products of the receptor. Namely, the ~120 kDa band corresponds to the mature, full-length holoreceptor containing N-linked complex oligosaccharides, while the ~75 kDa and ~50 kDa bands likely represent the A-subunit cleaved at different sites within the ectodomain, as previously described by Maruyama et al. [41] and Odo et al. [42]. The broader profile observed in tissue lysates, with bands ranging from ~100 kDa to 250 kDa, may reflect the presence of full-length, glycosylated, oligomeric forms of TSHR [43]. Such differences could arise from selective enrichment or processing of TSHR during vesicle formation, or from partial proteolysis and altered glycosylation occurring within the endosomal compartment. Although it has been suggested that the A-subunit of the TSHR dimer can be shedded and therefore found in the circulation [43], we did not detect TSHR in the supernatants remaining after EV enrichment. Therefore, we do not expect that the TSHR that was detected in our EV pellets originated from any possible protein contamination of the pellet. An observation that warrants further investigation is the significantly higher expression of the ~120 kDa TSHR band in EVs positive for CD63 in malignant compared to benign cases, which was not observed for the same marker in the total EV sample. Given the objectively small sample size in this analysis (*n* = 35), this observation should be interpreted with caution, but may point to potential differences in TSHR handling or incorporation into CD63-positive EVs that warrant further investigation.

Detection of vesicular Tg in EVs derived from the plasma of patients with thyroid nodules has not been previously investigated, and our results demonstrate that this protein can be detected in EV preparations using the dot blot method. Previous studies have demonstrated that EVs isolated from the plasma of patients with Hashimoto’s thyroiditis were positive for Tg, supporting our findings that Tg can be detected in circulating EVs [40]. A study by Huang et al. [17] analyzed vesicles derived from the urine of patients with thyroid carcinoma, and detected Tg in these EVs. While this study corroborates our results, it warrants further analysis as the glomerular filtration barrier does not permit the passage of structures larger than 4 nm, unless the barrier is compromised in the case of pathological kidney conditions [44].

In our Western blot analysis, the electrophoretic profile of vesicular Tg showed bands at the expected molecular weight for Tg monomers (330 kDa), as well as additional lower-molecular weight bands. It is somewhat possible that the lower MW bands may represent an unspecific binding of the primary monoclonal antibody. However, the Proteinase K treatment showed that the smaller bands are retained upon treatment, while it would be expected that they would be the primary degradation targets if originating from the contamination of plasma protein. Another explanation for detecting lower MW Tg might lie in the processing of the molecule during transcytosis inside the follicular cell. As a substantial fraction of Tg from the lumen of the follicle is endocytosed into the thyroid cell, it fuses with lysosomes and undergoes proteolytic cleavage to release T3 and T4, during which Tg may avoid complete degradation and might remain partially hydrolysed [45,46]. There is evidence that the transcytotic routes can intersect with recycling endosomal and MVB routes in polarized, epithelial cells, whereby endocytosed content can be secreted at the basolateral surface via MVB-associated secretory pathways, including fusion of MVBs with the plasma membrane [47,48,49,50]. We can thus hypothesize that this is how transcytotic vesicles containing partially degraded Tg may be secreted into the circulation. Additionally, the several postulated routes of Tg release into the bloodstream include vesicular secretion, thus providing conceptual support for our hypothesis [51].

The expression of vesicular Tg differed slightly between benign and malignant samples, for Tg bands at 330 kDa, in total EVs and CD63-positive EVs. Differentiated malignant thyroid tumors retain the expression of Tg [52], and the slightly higher expression we detected for vesicular Tg might reflect the higher secretion of Tg-loaded EVs. All recurrent samples, in which lymph node metastases were detected during follow-up, were also positive for vesicular Tg, suggesting a possibility for using vesicular Tg as a surrogate marker in TC monitoring; however, this finding should be interpreted with caution due to the limited sample size. This observation warrants further investigation in larger cohorts of recurrent cases, and also with a cohort of patients in complete remission that would serve as a necessary control.

Here, we must also address the fact that Tg detected in the EV preparation may arise from protein aggregates co-precipitated during dUC, an already recognized limitation of this method. However, one of the hypothesized mechanisms of Tg secretion into the circulation involves its release via vesicles [51], therefore we may be able to consider the Tg detected in our samples to be of predominantly vesicular origin. Still, circulating EVs can acquire a protein corona composed of abundant plasma proteins, meaning that some proteins detected in EV preparations may be surface-associated rather than truly intravesicular [53]. Our experiment with Proteinase K digestion to remove corona-bound proteins resulted in the loss of high MW Tg, pointing out that the expression of this Tg species may be originating from the protein corona. The fact that lower MW Tg was still present after digestion, may be pointing out at the possibility that the predominantly the truncated Tg species could be residing inside the vesicles. In an experiment where we probed EV-depleted supernatants from plasma, we did not detect Tg. Therefore, we can hypothesize that if any non-vesicular Tg is detected in our preparations, it may be arising from EV protein corona, rather than serum protein contamination. However, in any of these two issues that complicate the interpretation of vesicular Tg presence, the clinical context of detecting early DTC recurrence does not require that the precise origin of serum Tg is revealed if this approach can bypass the interference of anti-Tg antibodies that compromise immunometric assays. In a clinical scenario where a patient has anti-Tg antibodies and serum Tg levels are likely falsely low, detecting Tg by isolating Tg-containing vesicles may present a useful approach, regardless of potential contamination by small amounts of free serum Tg or protein corona-bound Tg. However, the origin of Tg warrants further elucidation, as understanding its source is important for interpreting these findings. It is interesting to note that free serum Tg levels did not correlate with EV Tg levels, except in the case of recurrent cases where a positive trend was observed; however, the very small sample size limits the interpretation of this result. When we looked at the Tg levels in cases grouped according to a differentiation score calculated according to histology and aggressiveness of the lesions, high MW Tg (330 kDa) showed a trend of rising with the rise in dedifferentiation and aggressiveness, especially in case EV Tg 330 kDa/CD63 where the correlation was significant. At the same time, the same trend was not observed for serum Tg. This result opens an interesting hypothesis on the differences in the secretion of vesicular Tg in less differentiated lesions.

Limitations of our study predominantly include the sample size used in some of the analyses, this also reflects one of the issues that arise when working with a scarce material such as extracellular vesicles. In EV research, experimental decisions often involve prioritizing which methods will be applied to specific samples, as sample volume is typically insufficient to perform all analyses on every sample. In our study this resulted in insufficient samples for the analysis of all selected proteins. In the sense of sample size, a key limitation of our study is the small number of patients with recurrent thyroid cancer, particularly those harboring anti-thyroglobulin antibodies. This limited sample size restricts the statistical power and generalizability of our findings within the subgroup most in need of novel biomarkers. Moreover, the absence of an appropriate control group, i.e., individuals without a thyroid gland but in complete remission, limits a more accurate assessment of thyroid-derived EVs and their specificity in the context of recurrence monitoring. A key strength of our study is combining cell line models and patient-derived plasma samples to explore biomarkers of thyroid-originating EVs. This strengthens the biological relevance of the results and translational potential of our findings. Furthermore, the use of multiple complementary methods, including NTA, Western blot, dot blot, ensured a reliable characterization of EVs and verification of their thyroid origin. By focusing on a clinically relevant question, particularly the potential role of EV-associated thyroglobulin in monitoring patients with anti-Tg antibodies, this study provides a novel perspective on overcoming current diagnostic limitations in thyroid cancer follow-up. To our knowledge, this is the first study to characterize thyroid-originating extracellular vesicles and their protein cargo in thyroid nodule patient plasma. Importantly, this study addresses a persisting clinical challenge in monitoring thyroid cancer recurrence in patients with anti-Tg antibodies. As a pilot investigation with an exploratory character, this study provides novel information that may serve as a foundation for pursuing an exciting approach to address an ever-burning issue in thyroid oncology.

## 4. Materials and Methods

### 4.1. Thyroid Cell Culture

Three thyroid cell lines—Nthy-Ori 3-1 (immortalized normal thyroid cells), TPC-1 (derived from papillary thyroid carcinoma) and OCUT2 (derived from anaplastic thyroid carcinoma)—were a kind gift from Dr. Pilar Santisteban’s laboratory, Institute for Biomedical Research Sols-Morreale, Madrid, Spain. Cells were cultured in Dulbecco’s Modified Eagle’s Medium—high glucose (Sigma Aldrich, Merck KGaA, Darmstadt, Germany supplemented with 10% (*v*/*v*) fetal bovine serum (FBS) (Sigma Aldrich, London, UK), 100 mM sodium pyruvate (Sigma Aldrich, Merck KGaA, Darmstadt, Germany), and antibiotic–antimycotic solution A5955 (10,000 U/mL penicillin, 10 mg/mL streptomycin and 25 µg/mL amphotericin B) (Sigma Aldrich, Merck KGaA, Darmstadt, Germany), in standard T25 flasks. After reaching 90% confluence cells were dissociated using TryplE Express (Thermo Fisher Scientific, Waltham, MA, USA) and plated in standard T75 flasks at 50% confluence. When cell confluence reached 80% the complete medium was replaced with FBS-free complete medium, in which the cells were cultured for 24 h. All phases of cell cultivation were carried out under 5% CO_2_ and 37 °C conditions. Cells were cultured at three different time points using independent batches of each cell line, in order to obtain biological replicates for EV analysis.

### 4.2. Patient Plasma and Clinical Data

Plasma samples from patients referred to surgical intervention due to the presence of thyroid nodules were obtained from the Clinic for Endocrine Surgery at the University Clinical Center of Serbia (UCCS). This research was approved by the Ethics Committee of UCCS, with a permit issued in September 2022 (No. 971/8). Samples were collected from 2022 until August 2025. Patients’ blood was collected preoperatively in EDTA-coated tubes, after which it was centrifuged at 400× *g* for 10 min at 4 °C, whereby blood cells were separated and supernatant was collected. The supernatant was then centrifuged at 2500× *g* for 15 min at 4 °C, for complete removal of platelets, and the resulting supernatant was collected and stored at −80 °C until further use.

The study included 47 benign and 35 malignant cases, out of which 5 were recurrent thyroid cancer cases. Patients were grouped according to pathohistological reports obtained from UCCS, from which all clinical and clinico-pathological data was also collected. This data included: age, gender, Serum Tg levels and Tg-Ab levels measured by enzyme immunometric tests, scores form ultrasound evaluation of thyroid nodules (EU-TIRADS) according to the European Thyroid Association guidelines for ultrasound malignancy risk stratification of thyroid nodules in adults [54], cytological diagnosis from fine needle aspiration biopsy (FNAB Cytology) according to the 2023 Bethesda System for reporting thyroid (Cytopathology Bethesda System for Reporting, [55]), tumor size, presence of lymph node metastasis (LNM), degree of tumor infiltration, extra-thyroid invasion, pT status, cancer stage according to the TNM staging system in accordance with the American Joint Committee on Cancer (AJCC) [56]. The degree of tumor infiltration (DI) was evaluated according to Basolo et al. [57] as follows: 1, totally encapsulated tumors; 2, non-encapsulated tumors without thyroid capsule invasion; 3, tumors with thyroid capsule invasion; and 4, tumors with extra-thyroid invasion. A differentiation score was calculated according to the histology of the lesion and its aggressiveness: 1, hyperplastic nodules; 2, thyroid follicular nodular disease (TFND); 3, follicular adenomas; 4, papillary thyroid cancer; 5, poorly differentiated thyroid cancer; 6, recurrent thyroid cancer. Cases with recurrent thyroid cancer had postoperative detection of locoregional (lymph node) metastasis and/or distant metastatic occurrence. One patient from the group was considered a case of persistent thyroid cancer as the Tg levels were detectable in the entire follow-up period upon total thyroidectomy.

### 4.3. Enrichment of EVs with Differential Ultracentrifugation

EVs from patient plasma and cell culture medium were enriched using the modified differential ultracentrifugation (dUC) method optimized by Théry et al. [58]. To prepare cell culture medium for dUC, the collected conditioned medium (10 mL) was centrifuged at 500× *g* for 5 min, at 4 °C, to remove the remaining whole cells, followed by centrifugation of the supernatant at 2000× *g*, 10 min, 4 °C to remove cell debris. Patient plasma (2 mL) was thawed at room temperature, and centrifuged at 5000× *g* for 10 min at 4 °C to remove the cryoprecipitate. After that, the supernatant was diluted 1:10 in sterile, particle-free, 50 mM phosphate-buffered saline (PBS) and centrifuged at 15,000× *g* for 45 min, 4 °C to remove larger vesicles and cellular debris. The processed cell culture medium and diluted patient plasma were then transferred into ultracentrifuge tubes, and volume was adjusted to 11 mL using sterile, particle-free PBS. Samples were centrifuged in a Beckman Coulter Optima L-90 ultracentrifuge (Beckman Coulter, Inc., Brea, CA, USA) using an SW 41 Ti rotor at 110,000× *g* for 2 h, 4 °C. Supernatants were stored until further use at −80 °C, while the pellet was resuspended in 100 µL of sterile, particle-free PBS and stored at −20 °C until further use. In the optimization phase, a protease inhibitor cocktail (Sigma Aldrich, Merck KGaA, Darmstadt, Germany) was added to plasma samples prior to dUC, in order to prevent the possible proteolytic degradation in the preparation. However, this addition had no influence on the protein analysis in Western blot, and was thus excluded in the final optimized protocol for isolation.

During the optimization of the EV enrichment stage, after dUC enrichment, several samples of EV preparations from cell culture medium and plasma EV were purified via Size Exclusion Chromatography (SEC), using Sepharose 2B (Sigma Aldrich, Merck KGaA, Darmstadt, Germany) columns, according to the manufacturer’s instruction. After the SEC step, EV preparations were concentrated using Vivaspin 500 Concentrator Polyethersulfone (Sartorius, Göttingen, Germany) columns, according to the manufacturer’s instructions. These samples were visualized by TEM which showed a significant loss of vesicles. Due to the insufficient amount of EVs needed for Western blot detection of EV proteins, the SEC purification method was later discarded.

### 4.4. Isolation of Protein from Adherent Cells and Thyroid Tissue

Proteins were isolated from adherent cell cultures and from tumor-adjacent normal thyroid tissue for the purpose of TSHR and Tg protein expression control. Adherent cells were dissociated using TriplE (Thermo Fisher Scientific, Waltham, MA, USA) and washed with PBS, followed by centrifugation at 500× *g* for 5 min. After dissociation, the cells were resuspended in a cold mixture of protein extraction buffer (20 mM Tris, 137 mM NaCl, 10% glycerol, 2 mM EDTA, 1% Nonidet P-40, pH = 8) and protease inhibitors P8340 (Sigma Aldrich, Merck KGaA, Darmstadt, Germany). Prepared samples were stored at −20 °C and were centrifuged at 12,000× *g* for 10 min at 4 °C prior to use, to remove any cell debris and other impurities. For the isolation of protein from the thyroid tissue, approximately 100 mg of tissue was resuspended in 1 mL mixture of cold extraction buffer (20 mM Tris, 137 mM NaCl, 10% glycerol, 2 mM EDTA, 1% Nonidet P-40, pH = 8) and protease inhibitor P8340. The samples were then homogenized using an LT QIAGEN homogenizer (QIAGEN, Hilden, Germany) at 50 Hz for 10 min, after which samples were centrifuged for 20 min at 12,000 rpm, 4 °C, in order to remove cell debris. After centrifugation, supernatant was collected and protein concentration was determined with BCA protein assay kit (Pierce, Rockford, IL, USA).

### 4.5. Nanoparticle Tracking Analysis (NTA)

Nanoparticle tracking analysis was used for determining the concentration and size distribution of EVs. This analysis was performed using the ZetaView PMX-420 QUATT and ZetaView software version 8.05.16 SP3 (Particle Metrix, Inning am Ammersee, Germany) calibrated according to the manufacturer’s instructions. Samples were measured at dilutions ranging from 1:200 to 1:1000 in sterile, particle-depleted 0.05 M PBS, pH 7.2, in order to obtain optimal particle count per frame, which was between 150 and 180 particles per frame. EV preparations were exposed to a blue laser (488 nm) and measurements were taken in scatter mode at up to 11 positions with sensitivity set at 78, shutter speed at 100 and a frame rate of 30 frames, during one cycle. Between each measurement a washing step with particle-depleted 0.05 M PBS, pH 7.2 was performed. Post-acquisition parameters were set to minimal area 10, maximal area 1000 and minimum brightness 30.

### 4.6. Transmission Electron Microscopy (TEM)

Preparation of samples for TEM analysis was performed according to the protocol of Kosanović et al. [59]. In brief, a 10 µL aliquot of each EV preparation was applied onto Formvar-coated copper grids by grid flotation for 45 min, at room temperature. Fixation with 2% formaldehyde for 10 min followed by washing three times with particle depleted 0.05 M PBS, pH 7.2, each for 2 min, was performed. Post-fixation was done using 2.5% glutaraldehyde, for 5 min, after which grids were rinsed with deionized water for 5 min. Grids were then air-dried at room temperature and TEM analysis was carried out on EVs isolated from conditioned medium and patient plasma, using a Philips CM12 electron microscope (Philips, Eindhoven, The Netherlands).

### 4.7. Dot Blot Analysis

The qualitative assessment of proteins in EVs was performed via dot blot analysis, in order to confirm their presence on the surface or inside EVs. For CD63 and TSHR detection, samples were directly applied onto the nitrocellulose membrane in a 10 µL volume, without adjusting for vesicle number. For detecting Tg in EVs samples, 5 min incubation at 100 °C in 1% SDS was performed in order to lyse the vesicle membrane and release the intravesicular cargo. Following sample application, membranes were blocked with 5% skimmed milk in TBST for 45 min, then washed 3 times in TBST. Membranes were incubated at room temperature for 1 h with appropriate antibodies—polyclonal anti-TSHR (Thermo Fisher Scientific, Waltham, MA, USA, Cat. No. PA5-116082) in 1:500 dilution, monoclonal anti-CD63 (TS63) (Invitrogen, Carlsbad, CA, USA, Cat. No. 10628D) in 1:1000 dilution and monoclonal anti-Tg SPTN-5 2805 (Medix Biochemica, Espoo, Finland, Cat. No. 100334) in 1:1000 dilution, after which membranes were incubated overnight at 4 °C. After incubating with primary antibodies, membranes were washed three times in TBST, followed by a 45 min incubation with secondary antibodies, using Goat Anti-Rabbit IgG (Vector Laboratories, Newark, CA, USA, Cat. No. BA-1000) for TSHR and Horse Anti-Mouse IgG (Vector Laboratories, Newark, CA, USA, Cat. No. BA-2000) for CD63 and Tg detection, both in 1:2000 dilution. Membranes were then washed 3 times in TBST and incubated with 1:1000 VECTASTAIN^®^ Elite^®^ ABC Kit, Peroxidase (Standard) (Vector Laboratories, Newark, CA, USA) solution for 30 min at room temperature. This was followed by 2 washes in TBST and a final wash in dH_2_O. Membranes were incubated with Enhanced Chemiluminescence (ECL) reagent (Thermo Fischer Scientific, Waltham, MA, USA) visualized using the ChemiDoc MP Imaging System (Bio-Rad Laboratories, Hercules, CA, USA), with exposure time of 1 h.

### 4.8. SDS-PAGE and Western Blot Analysis

For the detection of CD63 and TSHR proteins, 10% acrylamide gels were used, whereas 6% acrylamide gels were used for Tg detection. SDS-PAGE was conducted under reducing conditions, with samples denatured in reducing Laemlli buffer at 70 °C for 10 min. To ensure equal loading, samples were normalized to extracellular vesicle particle number measured by NTA, using the lowest measured particle concentration as the normalizer reference. Precision Plus Protein™ Kaleidoscope™ Prestained Protein Standards (Abcam, Cambridge, UK) were used for tracking protein size. A positive control—tumor-adjacent normal thyroid tissue lysate—was added to each run, to ensure the specificity of protein detection. Following SDS-PAGE, proteins were transferred onto the Amersham™ Protran 0.45 NC nitrocellulose Western blotting membranes nitrocellulose membrane (Cytiva, Freiburg, Germany) using a wet transfer method. Upon this step, the membranes were processed according to the previously described dot blot protocol—including blocking the membranes with 5% skimmed milk in TBST, incubation with primary and secondary antibodies, incubation with VECTASTAIN^®^ Elite^®^ ABC Kit and ECL reagent and visualized using the ChemiDoc MP Imaging System. After the target protein was detected in the analyzed samples, membranes were stripped and re-probed to detect CD63, loading control used for densitometric analyses. Stripping the membranes was performed using mild stripping protocol. Membranes were twice incubated with a stripping buffer (15% glycine, 1% SDS, 10% Tween 20, pH = 2.2) at room temperature for 7 min. This was followed by two wash outs in PBS for 10 min, at RT and two wash outs in TBST for 5 min, at RT. After stripping, membranes were blocked in 5% skimmed milk in TBST and treated per previously described dot blot/Western blot protocol. TotalLab TL120 1D v2009 software (Nonlinear Dynamics, Newcastle, UK) was used for the densitometric analysis of relative protein expression detected via Western blot.

### 4.9. Proteinase K Treatment of EVs

In order to remove the proteins from the protein corona of the EVs, Proteinase K pretreatment of EVs was performed. Briefly, EV samples were incubated with 2.5 ng/µL of Proteinase K (Sigma Aldrich, Merck KGaA, Darmstadt, Germany), for 5 min at 37 °C, after which the protease inhibitor cocktail (Sigma Aldrich, Merck KGaA, Darmstadt, Germany) was added in 1:100 ratio, to stop the digestion. Upon that, particle number was measured on NTA to assess that no significant vesicle degradation had occurred, and EVs were further analyzed by SDS-PAGE and Western blot as already described.

### 4.10. Statistical Analysis

The normality of the distribution was tested using Kolmogorov–Smirnov test or Shapiro–Wilk test. Differences between groups that were following the Gaussian (normal) distribution were assessed using Student’s *t*-test for two group comparisons, or one-way ANOVA for comparison between >2 groups. Correlation analyses were evaluated using Pearson’s correlation. Variables with continuous data were transformed logarithmically to fit the Gaussian distribution. For variables not distributed normally, Mann–Whitney U test was used for group comparison, and Spearman’s rho was assessed for correlation analyses. Receiver operating curve (ROC) analysis was used to assess diagnostic potential of selected markers and to determine the appropriate cut-off value for marker expression as a diagnostic test, and used to calculate sensitivity, specificity, positive predictive value (PPV), negative predictive value (NPV) and diagnostic accuracy (ACC). Results were considered significant if the *p*-value < 0.05. Statistical analysis was performed using RStudio (v2025.09.2, R Foundation for Statistical Computing, Vienna, Austria) and SPSS (SPSS 16.0, Chicago, IL, USA).

## 5. Conclusions

In this study we showed that the thyroid-specific membrane protein TSHR is present in EV-enriched preparations isolated from the plasma of patients with thyroid nodules and recurrent thyroid cancer. This opens a possibility for this marker to be employed as a target in constructing an immunoassay method for EV isolation. Electrophoretic profiles of TSHR and Tg in plasma EVs differed from tissue, which is a topic worth further investigation in order to gain more insight into a possible specific way of sorting proteins into vesicles, or, in the case of Tg, secretion of Tg inside vesicles. Intravesicular Tg presence has been detected in the EVs from plasma of patients with both thyroid nodules and patients with recurrent thyroid cancer who also harbor serum anti-Tg antibodies. These findings are consistent with the hypothesis that circulating EVs may harbor Tg and that in such form, Tg may be less accessible to Tg-Ab interference, and thus available for detection. This, therefore, opens an exciting research direction of a more detailed investigation into the hypothesis that vesicular Tg may be an alternative way of early recurrence detection. Due to the exploratory design and limited number of recurrent cases, these observations should be considered preliminary and warrant validation in larger prospective studies.

## Figures and Tables

**Table 1:** Clinical characteristics of the recurrent thyroid cancer cohort.

Case No.	1	2	3	4	5
Age (years)	26	72	68	90	35
Sex	F	M	F	F	F
TT	yes	yes	yes	yes	yes
Lymph nodal metastasis location	Central and lateral	lateral	Lateral	Lateral	Central
Histological type	PTC classical	PTC classical	PTC classical	PTC classical	PTC classical
pTNM	pT1bN1bMx	pT2pN1bM0	pT1bN1bM1	Unknown	pT2N1Mx
Tg (ng/mL)	25.03	n/a	25	38.6	24.69
Tg-Ab (IU/mL)	0	0	0	18.7	20
TSH (mUI/L)	0.27	0.04	0.008	1.53	0.062
Time in follow-up (months)	5	41	24	366	7
Persistent/Recurrent disease	Persistent	Recurrent	Recurrent	Recurrent	Persistent
Distant metastatic site	/	/	lungs	/	/

TT—total thyroidectomy, F—female, M—male, PTC—papillary thyroid cancer, Tg—thyroglobulin, Tg-Ab—antithyroglobulin antibodies, TSH—thyroid stimulating hormone, pTNM—pathological TNM stage.

**Table 2:** ROC analysis and the diagnostic performance of selected vesicular TSHR and Tg markers.

Marker	AUC	*p*-Value	Lower Bound	Upper Bound	Sensitivity %	Specificity %	PPV %	NPV %	ACC %
EV Tg 330 kDa	0.654	0.041 *	0.510	0.798	56.00	70.27	56.00	70.27	64.52
EV Tg 200 kDa	0.550	0.505	0.401	0.699	60.00	51.35	45.45	65.52	54.84
EV Tg 330 kDa/CD63	0.773	0.009 **	0.61	0.94	80.00	64.71	66.67	78.57	71.88
EV TSHR 120 kDa/CD63	0.721	0.029 *	0.554	0.888	71.43	61.90	55.56	76.47	65.71

EV—extracellular vesicle, TSHR—thyrotropin receptor, Tg—thyroglobulin. ROC—Receiver operating curve, AUC—area under the curve, PPV—positive predictive value, NPV—negative predictive value, ACC—diagnostic accuracy. *p*-value—statistical significance, * *p* < 0.05; ** *p* < 0.01.

## Data Availability

The raw data supporting the conclusions of this article will be made available by the authors upon request.

## Supplementary Materials

The following supporting information can be downloaded at: https://www.mdpi.com/article/10.3390/ijms27083510/s1.
