# Supplementary material for: Thyroid-Originating Extracellular Vesicles Harbor Thyroid-Specific Biomarkers with Potential Relevance for Thyroid Cancer Recurrence Detection
Source: Int J Mol Sci. 2026 Apr 14;27(8):3510. doi: 10.3390/ijms27083510 (PMC13116956; doi:10.3390/ijms27083510)
Supplement: Supplementary file 1 [file ijms-27-03510-s001.zip › ijms-4183195-supplementary.pdf]

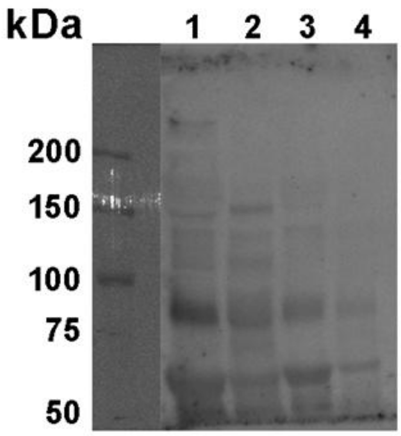

Figure S1. Western blot analysis of Tg presence in EV samples without Proteinase K treatment (1, 3) and with Proteinase K treatment (2, 4). Samples were treated with 2,5 ng/µL Proteinase K for 5min, at 37°C, and the treatment was stopped using Protease inhibitor cocktail.

Table S1. NTA measured EV number and particle sizes of samples pre- and post- Proteinase K treatment, before Western blot analysis.

		**Pre-treatment**	**After treatment**	**Fold**
**Sample 1**	Number	$5,9e^{10}$	$4,2e^{10}$	1,4
	Diameter (nm)	108	103,6	
**Sample 2**	Number	$2,6e^{10}$	$2,4e^{10}$	1,08
	Diameter (nm)	115,3	108,9	

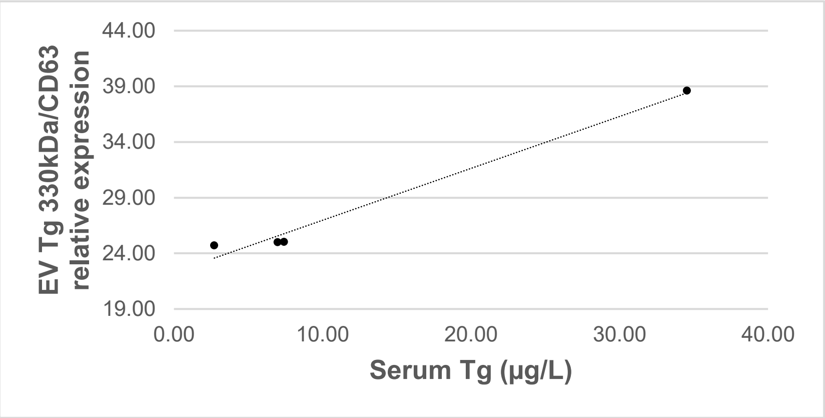

Figure S2. Correlation analysis of vesicular Tg (EV Tg 330kDa/CD63) and serum Tg expression levels in the cohort of recurrent thyroid cancer patients.

Table S2. Statistical correlation of densitometric measurements of TSHR protein expression and TSHR protein expression normalized on CD63 protein expression on the surface of EV enriched preparations from patient plasma with pathohistological features of patients with benign and malignant thyroid tumors.

	**Categories**	**TSHR 12OkDa**	**TSHR 70kDa**	**TSHR 50kDa**	**TSHR 120kDa/ CD63**	**TSHR 70kDa/ CD63**	**TSHR 50kDa/ CD63**
**Age (years)[a]**	20-90	R 0,025	R -0,331	R -0,062	R 0,017	R -0,300	R 0,048

		p 0,842	**p 0,037***	p 0,616	p 0,922	p 0,297	p 0,783
**Tg levels (ng/mL)** [a]		R 0,028<br>p 0,860	R 0,134<br>p 0,534	R 0,145<br>p 0,352	R -0,031<br>p 0,898	R 0,411<br>p 0,360	R 0,160<br>p 0,500
**TgAt levels (IU/mL)** [a]		R 0,059<br>p 0,695	R -0,266<br>p 0,180	R -0,229<br>p 0,122	R 0,054<br>p 0,806	R 0,395<br>p 0,332	R 0,046<br>p 0,835
**Tumor size (mm)**[a]	Range 5-128	R -0,022<br>p 0,866	R -0,429<br>**p 0,009****	R -0,151<br>p 0,237	R 0,078<br>p 0,678	R 0,297<br>p 0,374	R 0,038<br>p 0,837
**FNAB Cytology** [b]	I/II/III/IV/V/VI	ρ 0,220<br>p 0,156	ρ -0,130<br>p 0,537	ρ -0,035<br>p 0,826	ρ 0,414<br>p 0,055	ρ 0,256<br>p 0,579	ρ 0,380<br>p 0,081
**EU-TIRADS** [b]	1/2/3/4/5	ρ 0,207<br>p 0,141	ρ 0,212<br>p 0,260	ρ 0,105<br>p 0,457	ρ 0,401<br>**p 0,042***	ρ 0,152<br>p 0,719	ρ 0,303<br>p 0,132
**pT** [b]	pT1a/pT1b/pT2/pT3a/pT3b/pT4a/pT4b	ρ -0,197<br>p 0,357	ρ -0,582<br>**p 0,029***	ρ -0,144<br>p 0,503	ρ 0,375<br>p 0,229	N/A	ρ 0,007<br>p 0,982
**Degree of infiltration** [b]	A/B/C/D	ρ -0,009<br>p 0,967	ρ -0,287<br>p 0,319	ρ -0,030<br>p 0,887	ρ 0,148<br>p 0,630	ρ -0,866<br>p 0,333	ρ -0,170<br>p 0,579
**LNM** [c]	Absent/Present	p 0,508	p 0,131	p 0,263	N/A	N/A	N/A
**Degree of infiltration cut-off** [c]	A+B/C+D	p 0,615	p 0,629	p 0,976	N/A	N/A	N/A
**Extrathyroidal invasion** [c]	Absent/Present	p 0,838	p 0,172	p 0,866	N/A	N/A	N/A
**pT cut-off** [c]	pT1+pT2/pT3+pT4	p 0,624	p 0,077	**p 0,030***	N/A	N/A	N/A
**Stage** [c]	I/II	p 0,650	p 0,061	p 0,422	p 0,040	p 0,520	p 0,594

FNAB Cytology Categories correspond to the 2023 Bethesda system for reporting thyroid cytopathology categories: I – Non-diagnostic, II – Benign, III – Atypia of undetermined significance or follicular lesion of undetermined significance (AUS/FLUS), IV – Follicular neoplasm or suspicious for follicular neoplasm (FN/SFN), V – Suspicious of malignancy, VI – Malignant. EU-TIRADS Categories: 1 – Normal, 2 – Benign, 3 – Low risk, 4 – Intermediate risk, 5 – High risk. Degree of infiltration: A-totally encapsulated tumor, B-nonencapsulated tumor without thyroid capsule invasion, C - nonencapsulated tumors with thyroid capsule invasion, D - tumors with extrathyroidal invasion. LNM – lymph node metastasis, pT -pathological T status of tumor based on tumor size and invasion, p - p value, R - Pearson's correlation coefficient, ϱ - Spearman's rho. Stage calculated according to American Joint Committee on Cancer 8th edition of thyroid cancer staging. [a]Pearson's correlation, [b]Spearman's correlation, [c]Student's t test. *p<0,05; **p<0,01.

Table S3. Statistical correlation of densitometric measurements of Tg protein expression and Tg protein expression normalized on CD63 protein expression on the surface of EV enriched preparations from patient plasma with pathohistological features of patients with benign and malignant thyroid tumors.

	**Categories**	**Tg 330 kDa**	**Tg 200kDa**	**Tg 60kDa**	**Tg 330kDa/ CD63**	**Tg 200kDa/ CD63**	**Tg 60kDa/ CD63**
**Age (years)**[a]	20-90	R 0,214<br>p 0,095	R 0,162<br>p 0,207	R 0,053<br>p 0,704	R 0,308<br>p 0,086	R 0,277<br>p 0,124	R 0,011<br>p 0,956
**Tg levels (ng/mL)** [a]		R 0,057<br>p 0,725	R 0,058<br>p 0,722	R 0,030<br>p 0,864	R 0,019<br>p 0,941	R -0,192<br>p 0,446	R -0,267<br>p 0,337
**TgAt levels (IU/mL)** [a]		R 0,096<br>p 0,557	R 0,083<br>p 0,611	R 0,148<br>p 0,383	R 0,035<br>p 0,893	R -0,131<br>p 0,617	R 0,144<br>p 0,608
**Tumor size (mm)**[a]	Range 5-128	R 0,227<br>p 0,098	R 0,055<br>p 0,694	R 0,154<br>p 0,292	R 0,159<br>p 0,448	R 0,068<br>p 0,745	R 0,214<br>p 0,340
**FNAB Cytology** [b]	I/II/III/IV/V/VI	ρ 0,255<br>p 0,128	ρ -0,005<br>p 0,977	ρ -0,192<br>p 0,285	ρ 0,428<br>p 0,076	ρ 0,108<br>p 0,670	ρ 0,239<br>p 0,391
**EU-TIRADS** [b]	1/2/3/4/5	ρ 0,126<br>p 0,411	ρ 0,054<br>p 0,726	ρ -0,051<br>p 0,750	ρ 0,106<br>p 0,647	ρ 0,022<br>p 0,926	ρ 0,353<br>p 0,150
**pT** [b]	pT1a/pT1b/pT2/pT3a/pT3b/pT4a/pT4b	ρ -0,101<br>p 0,681	ρ -0,197<br>p 0,418	ρ -0,243<br>p 0,348	ρ -0,303<br>p 0,395	ρ 0,248<br>p 0,490	ρ 0,458<br>p 0,215
**Degree of infiltration** [b]	A/B/C/D	ρ 0,090<br>p 0,713	ρ 0,022<br>p 0,930	ρ -0,026<br>p 0,920	ρ -0,350<br>p 0,321	ρ 0,234<br>p 0,516	ρ 0,330<br>p 0,386
**LNM** [c]	Absent/Present	p 0,496	p 0,982	p 0,488	p 0,566	p 0,920	p 0,274
**Degree of infiltration cut-off** [c]	A+B/C+D	p 0,838	p 0,915	p 0,746	p 0,233	p 0,565	p 0,870

**Extrathyroidal invasion** [c]	Absent/Present	p 0,643	p 0,877	p 0,744	p 0,491	p 0,512	p 0,322
**pT cut-off** [c]	pT1+pT2/pT3+pT4	p 0,432	p 0,299	p 0,596	p 0,189	p 0,819	p 0,656
**Stage** [c]	I/II	p 0,676	p 0,390	p 0,805	p 0,888	p 0,606	p 0,212

FNAB Cytology Categories correspond to The 2023 Bethesda system for reporting thyroid cytopathology categories: I – Non-diagnostic, II – Benign, III – Atypia of undetermined significance or folllicular lesion of undetermined significance (AUS/FLUS), IV – Follicular neoplasm or suspicious for follicular neoplasm (FN/SFN), V – Suspicious of malignancy, VI – Malignant. EU-TIRADS Categories: 1 – Normal, 2 – Benign, 3 – Low risk, 4 – Intermediate risk, 5 – High risk. Degree of infiltration: A-totally encapsulated tumor, B-nonencapsulated tumor without thyroid capsule invasion, C - nonencapsulated tumors with thyroid capsule invasion, D - tumors with extrathyroidal invasion. LNM – lymph node metastasis, pT -pathological T status of tumor based on tumor size and invasion, p - p value, R - Pearson's correlation coefficient, ϱ - Spearman's rho. Stage calculated according to American Joint Committee on Cancer 8th edition of thyroid cancer staging. [a]Pearson's correlation, [b]Spearman's correlation, [c]Student's *t* test. *p<0,05; **p<0,01.

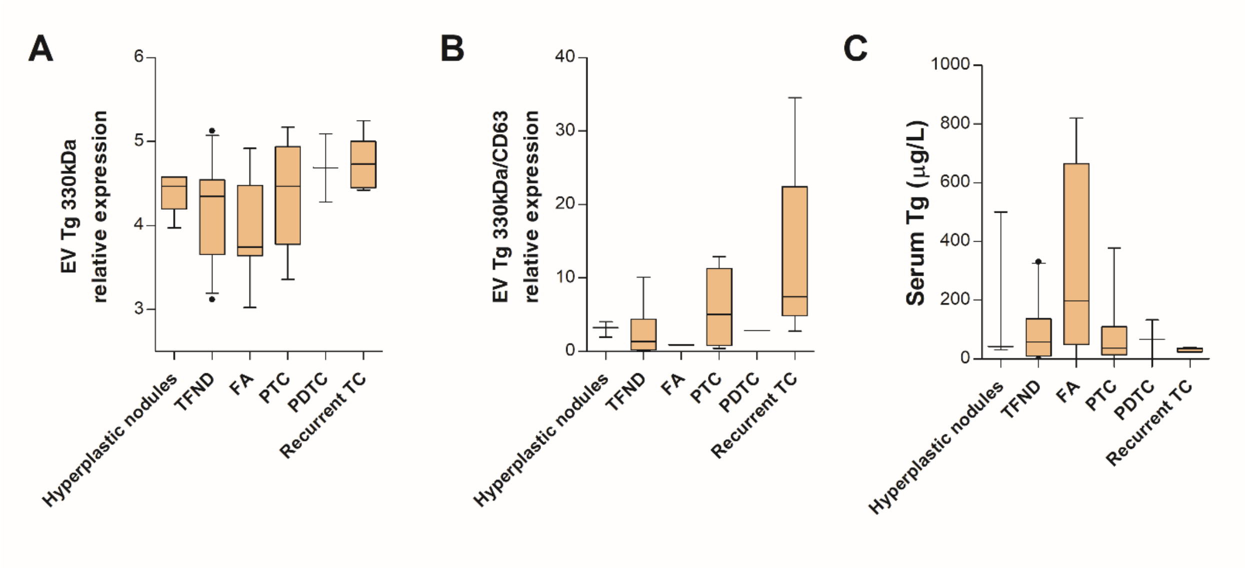

Figure S3. Expression of EV Tg330kDa, EV Tg 330kDa/CD63 and serum Tg in different thyroid nodules, classified according to histological species, dedifferentiation and aggressiveness. TFND-thyroid follicular nodular disease, FA-follicular adenoma, PTC-papillary thyroid cancer, PDTC-poorly differentiated thyroid cancer, recurrent TC-recurrent thyroid cancer.
